# Case Report: Spatially fractionated radiation therapy for local recurrence after prior radiotherapy in advanced right-sided small cell lung cancer with mediastinal lymph node metastases

**DOI:** 10.3389/fonc.2026.1829803

**Published:** 2026-05-11

**Authors:** Tian Tian, Chunhua Dai, Xin Yang, Leyao Liu, Jicheng Zhang, Wuyang Yang, Tao You

**Affiliations:** Tumor Center, Affiliated Hospital of Jiangsu University, Zhenjiang, China

**Keywords:** mediastinal lymph node metastasis of lung cancer, small cell lung cancer, spatially fractionated radiation therapy, lattice radiation therapy, peak-to-valley dose ratio

## Abstract

A 63-year-old male patient was admitted to the Affiliated Hospital of Jiangsu University on December 26, 2025, with a diagnosis of local recurrence in the mediastinal metastatic lymph nodes after radiotherapy, four years following the initial diagnosis of right-sided lung cancer. The patient was diagnosed with advanced small cell lung cancer of the right lung (cT3N3M1) and had previously received a combination of radiotherapy, chemotherapy, targeted therapy, and immunotherapy. Despite these treatments, the disease continued to progress, with resistance to both chemotherapy and immunotherapy, accompanied by widespread metastases. Palliative radiotherapy was administered for mediastinal lymph node enlargement starting July 2, 2025 (P_GTVn: 48 Gy/12 Fx). A follow−up CT scan on December 26, 2025, confirmed local in−situ recurrence of multiple mediastinal lymph nodes, accompanied by tracheal and superior vena cava compression and stenosis, indicating progressive disease (PD). From January 6, 2026, the patient underwent Spatially Fractionated Radiation Therapy (SFRT) targeted at the mediastinal metastatic lymph nodes, with a total dose of 30 Gy in 6 fractions, combined concurrently with atezolizumab immunotherapy. After five fractions of radiotherapy, follow−up computed tomography showed that the tumor volume decreased from 146 ml to 27 ml, representing a tumor regression rate of over 80%. Significant relief of left main bronchus compression was observed. No obvious radiation−related adverse events were noted during treatment or subsequent follow−up. Long−term efficacy requires further observation and evaluation.

## 1 Introduction

Lung cancer is the malignant tumor with the highest incidence and mortality worldwide (1). It also ranks first in both incidence and mortality among all malignant tumors in China (2). Statistical data demonstrate that the five-year survival rate of patients with advanced lung cancer remains below 20% in most countries (3). Advanced non-small cell lung cancer with mediastinal lymph node metastasis often causes persistent chest pain. Moreover, compression of adjacent anatomical structures, including the airway, esophagus, great vessels, and nerves, may lead to cough, dyspnea, hoarseness, and superior vena cava syndrome. These complications seriously affect both the survival and quality of life of affected patients. Radiotherapy is a commonly used approach for palliative symptom relief and quality-of-life improvement in patients with advanced lung cancer and mediastinal lymph node metastases. However, previous multimodal treatments, including radiotherapy, chemotherapy, targeted therapy, and immunotherapy, reduce the tolerance of normal tissues. In addition, the large tumor volume in advanced disease, together with the complex anatomy of the mediastinum and its proximity to critical organs, makes it difficult for conventional radiotherapy to achieve an optimal balance between sufficient tumor dose delivery and normal tissue protection (4). Spatially Fractionated Radiation Therapy (SFRT) represents a paradigm shift from conventional radiotherapy’s principle of uniform dose distribution. By adopting a “peak-valley” alternating dose pattern, SFRT induces synergistic tumor-killing mechanisms such as immune cell activation via the bystander effect and disruption of tumor microvasculature. This approach effectively achieves the dual objectives of local tumor control and normal tissue sparing, thereby significantly enhancing the precision and efficiency of radiation therapy (5). Lattice radiotherapy (LRT) is one of the main techniques of SFRT. This article reports a case of advanced right−sided small cell lung cancer with multiple metastases treated with SFRT for recurrent mediastinal lymph node metastases. The clinical outcomes are analyzed, aiming to provide a reference for the clinical management of similar cases.

## 2 Case presentation

### 2.1 Clinical data

The patient was a 63-year-old male with a several-year history of chronic obstructive pulmonary disease and hypertension, as well as a long-term smoking history. Bronchoscopy with transbronchial lung biopsy was performed on December 28, 2021. Immunohistochemical staining results were as follows: AE1/AE3 (+), CK7 (–), Napsin-A (–), CD56 (+), Syn (+), CgA (–), P40 (–), P63 (–), CK5/6 (–), Ki-67 (75%+), TTF-1 (+). Combined with hematoxylin and eosin (HE) staining sections, the morphological and immunophenotypic features were consistent with small cell lung cancer. The pathological report (No. I210018) of the right lung biopsy tissue confirmed the diagnosis of small cell cancer. Based on comprehensive imaging and pathological findings, the patient was diagnosed with stage IIIA small cell lung cancer of the right lung.

From January 12 to June 25, 2022, the patient received six cycles of chemotherapy with etoposide 0.1 g on days 1–4 and cisplatin 60 mg on days 1–2, combined concurrently with atezolizumab 1200 mg, followed by maintenance immunotherapy with atezolizumab 1200 mg. A follow-up computed tomography (CT) scan on June 8, 2024, showed an enlarged soft-tissue mass in the subcarinal region of the mediastinum compared with prior imaging, suggestive of mediastinal lymph node metastasis and consistent with progressive disease (PD). From June 9 to November 12, 2024, the patient received an additional six cycles of combination therapy consisting of etoposide 0.1 g on days 1–4, cisplatin 40 mg on days 1–3, and immunotherapy. The initial efficacy evaluation was partial response (PR), which was subsequently re-evaluated as stable disease (SD).

On January 19, 2025, the disease was assessed as PD. From January 22 to May 11, 2025, the regimen was adjusted to five cycles of chemotherapy with nab-paclitaxel 400 mg and carboplatin 500 mg, combined with atezolizumab 1200 mg. An interim evaluation on March 22, 2025, showed PR. However, PD was confirmed after the final cycle on May 11, 2025. Targeted therapy with anlotinib was initiated on May 14, 2025 (12 mg, subsequently reduced to 8 mg), followed by seven cycles of atezolizumab 1200 mg from June 5 to October 29, 2025. Palliative radiotherapy was delivered to the enlarged mediastinal lymph nodes starting July 2, 2025 (P_GTVn: 48 Gy/12 Fx). On October 7, 2025, the disease was evaluated as SD. In November 2025, magnetic resonance imaging(MRI) revealed a suspected occupying lesion in the pancreatic head. Subsequent PET-CT demonstrated a soft-tissue mass in the pancreatic head (with ill-defined borders adjacent to the descending duodenum) with increased FDG uptake, suggestive of malignant metastasis. Palliative radiotherapy for the pancreatic metastatic lesion was started on November 22, 2025 (P_GTV: 42 Gy/14 Fx).

A follow-up CT scan on December 26, 2025, showed multiple enlarged and partially fused mediastinal lymph nodes with significant progression compared with the previous scan on October 7, 2025, suggestive of metastatic disease, accompanied by compression and stenosis of the trachea and superior vena cava. The disease status was assessed as PD. Starting December 27, 2025, the chemotherapy regimen was adjusted to liposomal irinotecan 86 mg on days 1 and 14, plus nedaplatin 40 mg on days 1–3, combined with atezolizumab 1200 mg.

### 2.2 Physical examination and auxiliary examinations

Physical Examination: The patient was alert and cooperative. No enlarged superficial lymph nodes were palpable. Bilateral breath sounds were decreased, with no obvious dry or moist rales. Cardiac rhythm was regular, and no pathological murmurs were detected on auscultation. The abdomen was flat and soft, with no tenderness, rebound tenderness, or palpable masses. No edema of the lower extremities was noted.

**Figure 1:**
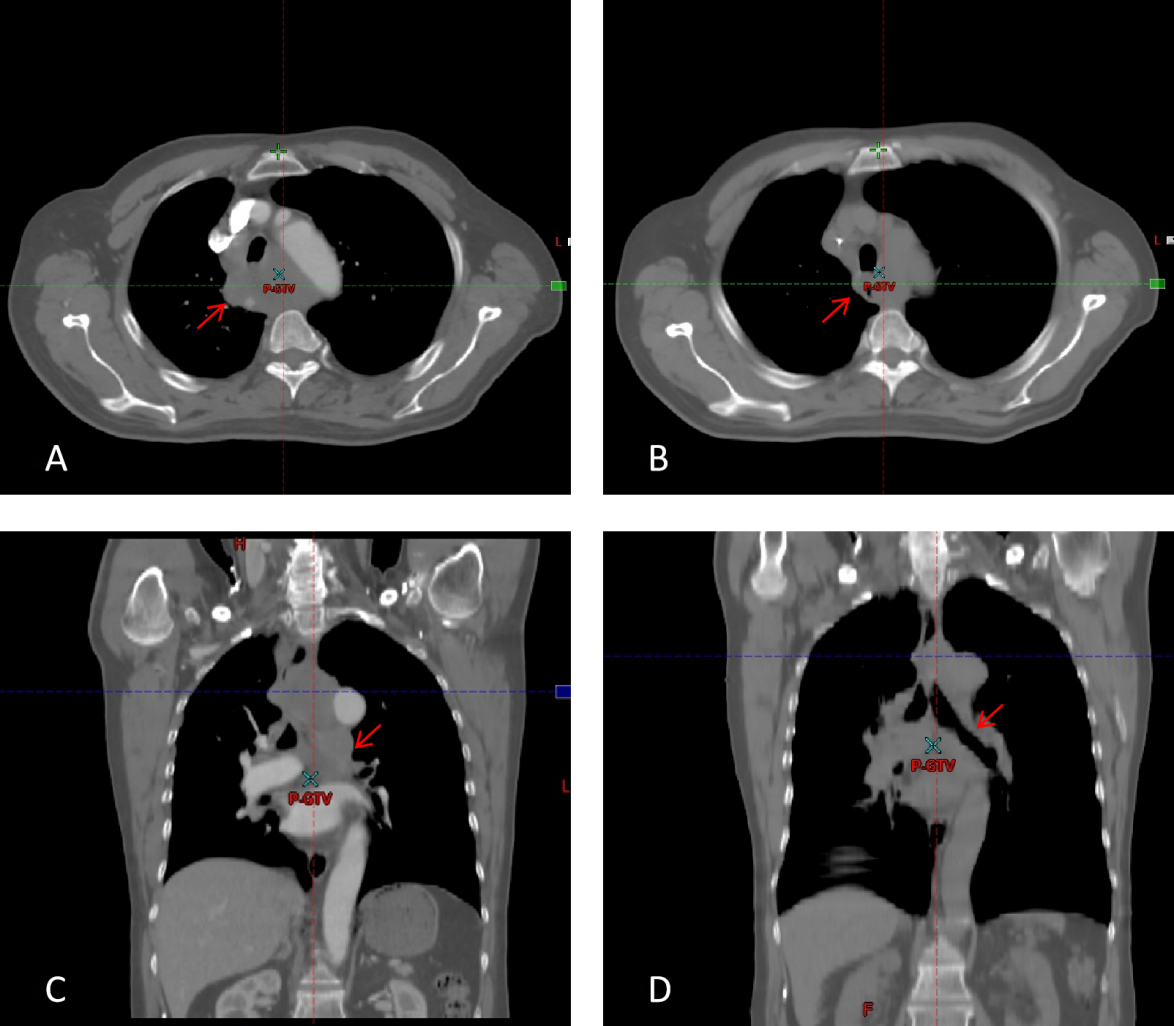
Cross-sectional and coronal CT images before and after treatment. **(A, C)** axial and coronal images before treatment, respectively. **(B, D)** axial and coronal images after 5 fractions of SFRT, respectively.

Auxiliary Examinations: CT imaging performed on January 4, 2026 (**Figures 1A, C**) demonstrated multiple enlarged mediastinal lymph nodes with partial coalescence. The largest lesion measured approximately 6.1 cm × 4.2 cm in cross-section. Although slightly decreased in size compared with prior imaging, the lesions were highly suggestive of metastatic disease, accompanied by compression and stenosis of the trachea and superior vena cava.

### 2.3 Treatment

LRT was initiated on January 6, 2026, for metastatic mediastinal lymph nodes, with a dose of 30Gy/6Fx. This radiotherapy plan was designed using the Varian Eclipse 13.6 treatment planning system, with Volumetric Modulated Arc Therapy (VMAT) and three non−coplanar arcs. Treatment was delivered using a Varian TrueBeam STx linear accelerator (Varian, USA). Real−time monitoring was performed during treatment by integrated thermo−optical surface monitoring and X−ray image guidance system (BrainLab, Germany).

**Figure 2:**
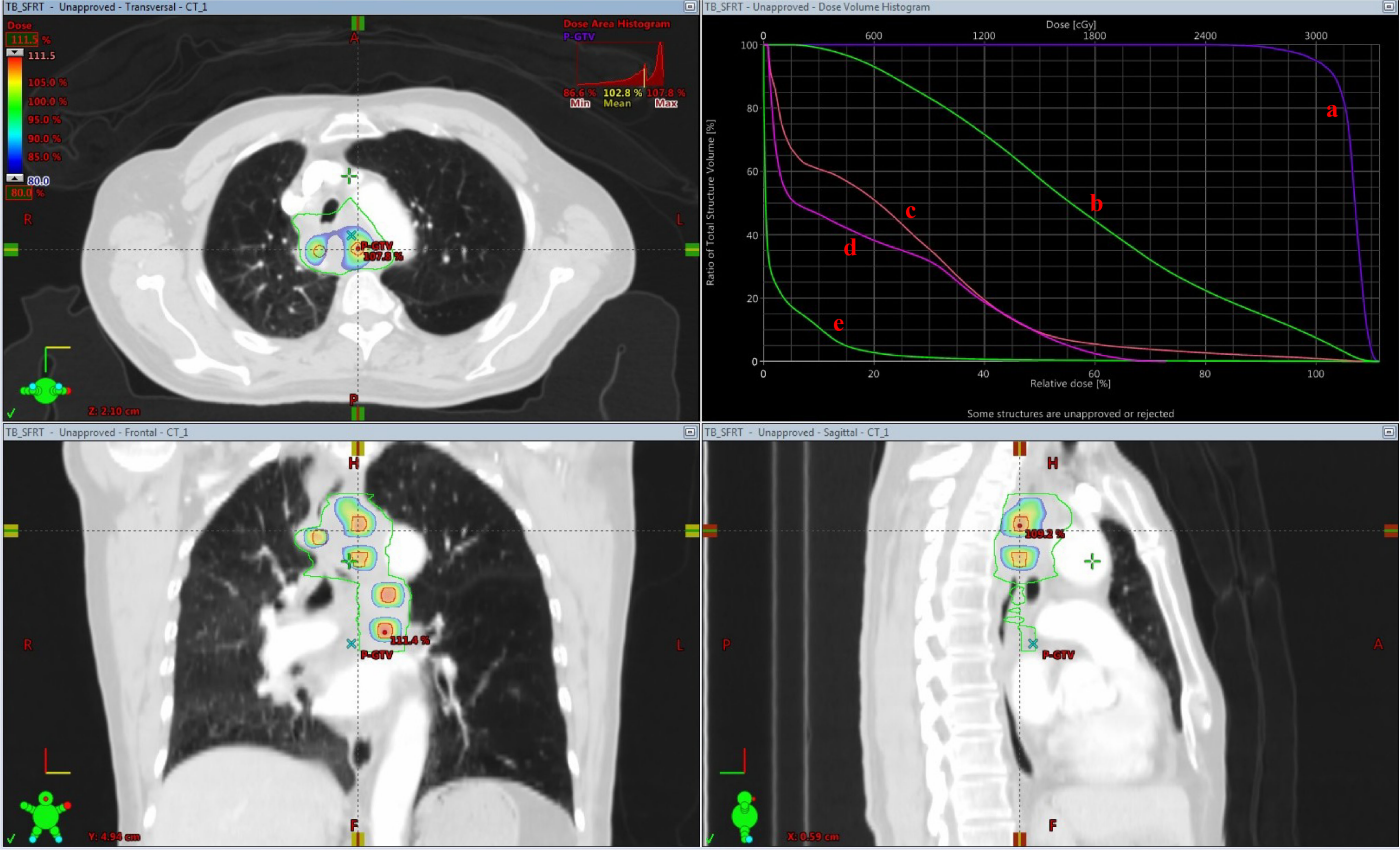
Dose distribution in target volume: the relative positions of high-dose regions (peaks) and GTV on axial, sagittal, and coronal CT images, and the corresponding DVH curves: Curve **(a)** DVH curve of the planning target volume (PTV), namely the SFRT target volume; curve **(b)** DVH curve of the entire solid tumor; curve **(c)** DVH curve of the esophagus; curve **(d)** DVH curve of the trachea; curve **(e)** DVH curve of the body.

**Figure 3:**
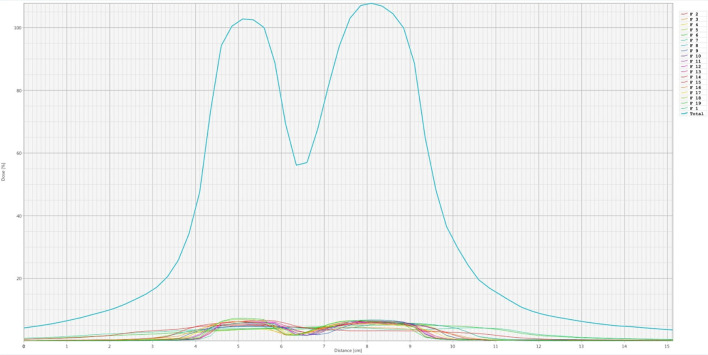
Ratio curve of high−dose to low−dose regions within the GTV, used for the calculation of the PVDR.

Target volume dose distribution and dose−volume histogram (DVH) of the patient treated with LRT are presented in **Figure 2**. It directly displays the locations of high-dose regions (peaks) and gross tumor volume (GTV) on axial, sagittal, and coronal CT images. In the three sectional views, warmer color tone within the target volume indicates an increased radiation dose in this region. The red circular rings represent the SFRT sub-target volumes; the red dose color wash indicates the region receiving ≥100% prescription dose, and the surrounding blue dose color wash represents the 50% isodose line. The dose decreases rapidly over a short distance (1.0–1.5 cm) from the central red target region to the peripheral blue dose color wash. In the DVH graph, the horizontal axis represents dose percentage, and the vertical axis represents the volume percentage. The specific DVH curves of the relevant organs are shown in **Figure 2**. Meanwhile, **Figure 3** shows the high-to-low dose ratio curve within the GTV, which can be used to calculate the peak-to-valley dose ratio (PVDR). The horizontal axis denotes distance, and the vertical axis denotes dose percentage. Two dose peaks corresponding to core target volumes both exceeding 100%, with an interval of more than 3 cm. The valley dose is below 60%, indicating a rapid dose fall−off and steep peak−to−valley dose ratio between targets. The curve margin demonstrates that the dose to surrounding normal tissues rapidly declines to below 10%.

The patient exhibited favorable overall tolerance throughout treatment, with no grade ≥3 acute toxicities observed.

### 2.4 Outcome and follow-up

Follow-up CT performed on January 10, 2026 (**Figures 1B, D**), after 5 fractions of treatment, showed that multiple enlarged mediastinal lymph nodes were reduced in size compared with the previous scan on January 3, 2026. The largest lesion measured approximately 3.1 cm × 2.2 cm in cross-section. Mild compression of the trachea, esophagus, and superior vena cava was noted, representing marked improvement compared with prior imaging.

The patient achieved a favorable regression of mediastinal lymph node metastases. After 5 fractions of SFRT, the mediastinal tumor volume decreased from 146 ml to 27 ml, representing a volume reduction rate of over 80%. Long-term efficacy remains to be further evaluated during continued follow-up.

## 3 Discussion

Conventional radiotherapy typically delivers a homogeneous dose distribution within the target volume, which often leads to considerable injury to surrounding normal tissues. In contrast, SFRT achieves a highly heterogeneous dose distribution characterized by alternating high-dose “peaks” and low-dose “valleys” across the tumor region. The high-dose peaks directly ablate tumor cells, whereas the low-dose valleys spare adjacent normal tissues and organs, thus improving local tumor control while reducing radiation-related toxicity. Owing to the steep dose fall-off gradient, radiocytotoxic effects are mainly confined to the tumor region, allowing effective protection of neighboring critical organs and healthy tissues (6–8). Based on these advantages, SFRT is currently widely used in preoperative neoadjuvant therapy, palliative care, and the management of metastatic and recurrent malignancies. Its clinical applications cover a variety of solid tumors, including head and neck cancer, lung cancer, breast cancer, gynecologic neoplasms, and sarcomas (9). Currently, the main technical modalities of SFRT in clinical practice include Grid Radiation Therapy (GRID), LRT, Microbeam Radiation Therapy (MRT), and Minibeam Radiation Therapy (MBRT). Developed on the basis of GRID, LRT further improves the conformality of dose distribution within the target volume and achieves a more favorable PVDR. These features enhance the protection of surrounding normal tissues, especially in the treatment of large, unresectable, or metastatic tumors (10). A study by Amendola et al. applied LRT in 12 patients with advanced non-small cell lung cancer (NSCLC) and demonstrated significant tumor regression, improved local control, minimal radiation toxicity, and a certain prolongation of overall survival (11). Furthermore, pooled analyses have demonstrated that LRT presents favorable safety and efficacy profiles, with complete remission achieved in some patients. Marked symptomatic improvement was observed in palliative patients, and local control rates in high-dose regions reached up to 90% (12). As a key parameter for LRT optimization, PVDR effectively reflects the degree of dose heterogeneity in radiotherapy plans. Several studies have suggested that a high PVDR is more conducive to activating multiple biological mechanisms, including immunogenic cell death, while preserving normal blood vessels and the tumor microenvironment (13). However, excessively elevating PVDR alone is insufficient to improve therapeutic efficacy. This may be attributed to the potential damage to immune cells in the low-dose valley regions, the weakened bystander effect, and even increased toxicity to adjacent normal tissues (14, 15).

This study reports a case of advanced small cell lung cancer in the right lung. The patient had previously received a combination of radiotherapy, chemotherapy, targeted therapy, and immunotherapy. Despite multiple treatment interventions, the disease continued to progress, accompanied by the development of chemotherapy and immunotherapy resistance. The patient presented with extensive metastases, advanced disease stage, and a poor prognosis. Local recurrence was observed in the primary lesion, accompanied by a large metastatic mediastinal lymph node measuring more than 6 cm, which was adjacent to multiple critical organs and tissues, consistent with the indications for SFRT. Notably, the patient had undergone palliative radiotherapy for metastatic mediastinal lymph nodes 6 months prior (prescribed dose: 48 Gy/12 Fx), during which the radiation doses to organs at risk, including the trachea, esophagus, and spinal cord, had already approached their tolerance limits. Although early treatment response was achieved, subsequent chemotherapy and immunotherapy failed to provide sustained disease control. Therefore, LRT was delivered to the metastatic mediastinal lymph node with a prescribed dose of 30Gy/6Fx. In current clinical practice, the recommended parameter settings for LRT planning typically include a microsphere diameter of 0.5–1.5 cm and an inter-microsphere spacing of 2.0–5.0 cm (16) while critical organs at risk (OARs), blood vessels, and bones are deliberately spared. In the treatment planning, the lattice target volume was spherical with a diameter of 1 cm. The interval between adjacent spheres, as well as their distances to the solid tumor margin, trachea, and esophagus, were all ≥1.5 cm. The mean prescription dose (Dmean) within the high-dose spherical volume (1 cm diameter) reached 104.8% of the prescribed dose, with the maximum dose (Dmax) hitting 113%. In contrast, the lowest dose region within the P_GTV achieved a mean prescription dose of 23.8%. The spherical centers were spaced 2.0 cm apart, maintaining a minimum distance of >1 cm from the trachea, major blood vessels, and esophagus. The resulting PVDR was calculated to be 4.7. Throughout the entire treatment course and subsequent follow-up, no significant radiotherapy-related adverse events were observed, demonstrating favorable safety profiles. Post-treatment CT imaging revealed a substantial reduction of the mediastinal lymph node lesions (>80% reduction in volume) with marked alleviation of left main bronchial obstruction. Notably, the combination of LRT with atezolizumab immunotherapy yielded promising therapeutic outcomes, with a confirmed PR per post-treatment evaluation. In a meta-analysis by Iori et al. encompassing 20 patients with bulky tumors (>4.5 cm in diameter) treated with LRT, the objective response rate at 3 months was approximately 79%, with a median target volume reduction of 54%. Furthermore, no acute or late adverse effects of grade ≥3 were reported in any of the patients (17). Collectively, these findings substantiate LRT as a safe and effective therapeutic option for palliative care, particularly in patients with large-volume disease—a conclusion that aligns well with the favorable treatment outcomes observed in our case.

In the management of patients with malignant tumor lymph node metastasis, radiotherapy plays a pivotal role as a core therapeutic modality. However, mediastinal lymph nodes are closely adjacent to major blood vessels, the heart, and bronchi, posing a substantial risk of radiation-induced toxicity. Thus, particular caution is warranted when applying conventional radiotherapy techniques (18). Although stereotactic body radiation therapy (SBRT) has shown promising local control efficacy in the treatment of mediastinal and hilar metastatic lymph nodes (19), strict adherence to constraints on radiation dose and target volume remains imperative (20). Studies have indicated that the antitumor efficacy of SFRT stems not only from the physical eradication of tumor cells but, more importantly, from its unique biological mechanisms—including the radiation-induced bystander effect, immunomodulatory effects, and microvascular alterations. These mechanisms act synergistically: the bystander effect indirectly suppresses tumor cells in non-targeted “valley regions” and distant sites, thereby amplifying localized cytotoxicity; the immunomodulatory effects activate systemic antitumor immune responses; and the microvascular effects disrupt the tumor blood supply, undermining its survival basis (5, 13). Building on these mechanisms, SFRT effectively achieves multiple therapeutic goals, including tumor ablation, preservation of adjacent normal organs, palliative symptom relief, reversal of immunotherapy resistance, and enhancement of treatment response rates. Consequently, it contributes to improved local tumor control, better prognosis, enhanced treatment safety, reduced radiation-related toxicities, and ultimately, improved patient quality of life. In the present case, the patient experienced *in situ* recurrence of mediastinal lymph nodes following prior high-fraction radiotherapy (48 Gy/12 Fx), with surrounding normal organs having received radiation doses approaching their tolerance limits. Owing to reduced normal tissue tolerance after prior chemotherapy and an immunotherapy-resistant state, conventional clinical strategies offered limited therapeutic benefit. Notably, a thoracic CT scan performed immediately after 5 fractions of LRT showed effective control of the mediastinal lymph node lesion, with the tumor volume decreasing from 146 mL to 27 mL—a reduction exceeding 80%. Compression of the left main bronchus was significantly alleviated, yielding outcomes that exceeded expectations. Given that the mediastinal metastatic lymph nodes represented post-radiotherapy recurrence, SFRT alone would be unlikely to achieve such pronounced results. Considering the concurrent administration of atezolizumab immunotherapy, it is plausible that SFRT-induced reversal of immunotherapy resistance re-sensitized the previously resistant tumor to immune activation. This likely generated a synergistic effect between radiotherapy and immunotherapy, ultimately driving rapid tumor regression and achieving remarkable therapeutic efficacy.

This case study explores the application of SFRT in the management of lung cancer with mediastinal lymph node metastasis, focusing on its technical advantages, clinical indications, and therapeutic outcomes. Currently, standardized definitions for key dosimetric parameters—including PVDR, peak dose, and valley dose—have not yet been established in existing research. As a result, radiotherapy planning remains largely dependent on clinical experience, lacking universally accepted guidelines and protocols. The present case suggests that SFRT may exert a synergistic effect with immunotherapy by reversing immune resistance pathways, achieving unexpectedly favorable short-term therapeutic outcomes. However, the underlying mechanisms are likely regulated by multiple factors, and the generalizability of these findings requires further validation through large-scale multicenter studies. Furthermore, personalized strategies for combining SFRT with immunotherapy—such as optimal dosing schedules and treatment sequencing—as well as the potential benefits of concurrent administration with other antitumor agents, require systematic investigation in future research.

## Data Availability

The data analyzed in this study is subject to the following licenses/restrictions: The data supporting the conclusions of this article is available from corresponding author on reasonable request. Requests to access these datasets should be directed to Tao You, ty_zj@ujs.edu.cn.
